# Mapping the global research output of Traditional Chinese Medicine in the treatment of metabolic dysfunction-associated steatotic liver disease: a comprehensive bibliometric analysis based on multiple databases (2000–2025)

**DOI:** 10.3389/fmed.2026.1754639

**Published:** 2026-03-25

**Authors:** Da Wang, Mengwei Li, Rongting Zhao, Hui Wang, Hang Chen, Minshan Huang, Yingxue Shen, Lanqing Ma

**Affiliations:** The First Affiliated Hospital, Yunnan Institute of Digestive Disease, First-Class Discipline Team of Kunming Medical University, Kunming, China

**Keywords:** Traditional Chinese Medicine, metabolic dysfunction-associated steatotic liver disease, bibliometrics, network pharmacology, gut microbiota, multi-omics, bioactive components

## Abstract

**Objective:**

The application of Traditional Chinese Medicine (TCM) in managing metabolic dysfunction-associated steatotic liver disease (MASLD) has attracted growing interest in the academic community. However, a comprehensive quantitative analysis of its therapeutic effects on MASLD remains lacking. This research seeks to analyze the current landscape and research trends in TCM treatment for MASLD from 2000 to 2025, thereby offering insights and guidance for future investigations.

**Methods:**

We extracted relevant literature published from 2000 to 2025 from the Web of Science and Scopus databases. The collected data were then processed, analyzed, and graphically represented using Origin, R software, VOSviewer, and CiteSpace.

**Results:**

A total of 889 articles (from Web of Science) and 983 articles (from Scopus) were included in the study. From 2000 to 2025, the number of publications demonstrated a consistent upward trend, with particularly significant growth occurring between 2019 and 2022, followed by a slight decline in 2023. China is the leading country in publication output and holds a pivotal position in fostering international research collaboration. The *Journal of Ethnopharmacology* is the most productive journal in this area. Current research hotspots primarily center on exploring the potential mechanisms of TCM interventions for MASLD, their clinical applications, and the identification and utilization of bioactive components. Mechanistic studies mainly focus on “regulating lipid metabolism,” “anti-inflammatory and anti-fibrotic effects,” and “reversing gut dysbiosis.” Notably, the biotransformation role of gut microbiota is gradually emerging as a key research area.

**Conclusion:**

As a traditional medical system with multi-target effects and holistic regulatory advantages, TCM is gaining increasing attention in the management and prevention of MASLD. This study provides a comprehensive overview and evaluation of current research on TCM in the treatment of MASLD, providing valuable insights for future studies in this field.

## 1 Introduction

Metabolic dysfunction-associated steatotic liver disease (MASLD), previously known as non-alcoholic fatty liver disease (NAFLD), is diagnosed by the presence of hepatic steatosis along with one or more of the following conditions: overweight or obesity, diabetes mellitus, or signs of metabolic dysfunction (1). The disease spectrum of MASLD include a series of progressive liver-related pathologies, progressing from isolated steatosis to metabolic dysfunction-associated steatohepatitis (MASH), advanced fibrosis, cirrhosis, and ultimately hepatocellular carcinoma (HCC) (2). Patients with MASLD not only have a markedly elevated risk of adverse liver-related outcomes, including cirrhosis, hepatic failure, and HCC, but also experience an increased incidence of extra-hepatic complications and higher all-cause mortality, such as from cardiovascular disorders, chronic renal disease, and extra-hepatic neoplasm-like colorectal cancer (3, 4). As the prevalence of obesity, diabetes, and metabolic syndrome continues to rise globally, MASLD has emerged as a significant public health issue and is now recognized as the leading form of chronic liver disease worldwide (5). Recent epidemiological evidence shows that the global prevalence of MASLD rose from 25.3% (1990–2006) to 38.2% (2016–2019), reflecting a nearly 50% growth in the worldwide burden of MASLD over the past three decades (6). MASLD is projected to become the primary reason for liver transplants by 2030 (7). It is evident that MASLD imposes a substantial burden, significantly impairing patients’ quality of life and incurring considerable social and economic expenses. Currently, standardized drug treatment plans for MASLD remain lacking (8). Lifestyle interventions, such as calorie restriction and physical exercise, constitute the cornerstone of MASLD management; however, their therapeutic efficacy is limited by poor long-term patient compliance (9, 10). Therefore, actively exploring effective interventions for MASLD can contribute to improved patient outcomes and reduce the public health burden.

With a history of development and refinement spanning thousands of years, Traditional Chinese Medicine (TCM) has achieved widespread recognition and application across numerous countries such as China, Japan, South Korea, and India (11). Based on the principles of “holistic view” and “dialectical treatment,” TCM emphasizes individual variability and the multifactorial origins of diseases, demonstrating significant potential in the management of MASLD (12). Extensive research has demonstrated that TCM can ameliorate MASLD via various pathways, such as promoting lipid metabolism, mitigating oxidative stress, reducing inflammatory responses, alleviating hepatic fibrosis, and regulating gut microbiota. For example, Yinchen Linggui Zhugan decoction has been shown to reduce hepatic lipid buildup in rats fed a high-fat diet (HFD) by activating the sirtuin 1 (SIRT1) signaling pathway and remodeling gut microbiota composition (13). Moreover, the therapeutic potential of TCM extracts is also gradually being explored and recognized in scientific and clinical research. Ginseng extracts, such as ginsenoside Rg2, have been demonstrated to ameliorate hepatic steatosis, improve glucose tolerance and enhance insulin sensitivity in HFD-induced mouse models, through a mechanism dependent on SIRT1 (14). *In vivo* experiments have demonstrated that berberine (BBR) effectively reduces triglyceride (TG) synthesis and alleviates liver steatosis through activation of the AMP-activated protein kinase (AMPK) signaling pathway (15). Moreover, BBR has been shown to suppress inflammation responses by decreasing immune cell infiltration and downregulating the expression of pro-inflammatory mediators (16). In an 18-week, placebo-controlled phase 2 clinical study, berberine ursodeoxycholate was found to significantly reduce hepatic fat content among patients with both MASH and type 2 diabetes mellitus (17). It can be seen that TCM and its extracts have provided novel therapeutic strategies for MASLD through multi-target and multi-pathway characteristics. Regrettably, although numerous studies have explored TCM for treating MASLD, this field still lacks a systematic analysis of research hotspots and frontier trend. Therefore, it is necessary to systematically review current research progress on TCM treatment for MASLD to provide researchers with a comprehensive overview of the fields’ research status and developmental trends.

Bibliometrics is a quantitative approach that analyzes scientific literature through mathematical and statistical methods, revealing the current state, research frontiers, and developmental trends within specific academic fields (18). Given the significant role of TCM in MASLD research, employing this method facilitates a systematic review of research progress, identifies gaps, and provides direction and theoretical support for future studies. Therefore, this study will comprehensively evaluate the research on TCM for MASLD treatment through bibliometric analysis, aiming to promote in-depth advancement in this area and further enhance the utilization of TCM in MASLD therapy.

## 2 Materials and methods

### 2.1 Data sources and search strategy

We searched the Web of Science Core Collection (WoSCC) and Scopus databases to retrieve relevant studies. The literature was screened according to the following inclusion criteria:(1) search terms and strategies are presented in Table 1, (2) the literature was issued between January 1, 2000 and July 31, 2025, (3) only studies published as ‘REVIEW’ or ‘ARTICLE’ were included, and (4) the publication language is English. Exclusion criteria were as follows: (1) literature types such as conference papers, newspapers, patents, achievements, health and popular science literature, etc., (2) studies that did not primary focus on the application of TCM, and (3) publications that did not demonstrate the use of TCM in the context of MASLD. The publications retrieved from WoSCC were saved in plain text format, whereas those obtained from Scopus were saved in CSV files containing complete information, including author information, title, publication year, source title, volume, issue, paper range, DOI, literature information, institutional affiliation, ISSN, funding details, and cited references.

**Table 1:** Diagram of retrieval strategy.

Databases	Search strategy
WoS core collection	TS = (NAFLD OR “non alcoholic fatty liver disease” OR “nonalcoholic fatty liver disease” OR “non-alcoholic fatty liver disease” OR “nonalcoholic fatty liver” OR MASLD OR “metabolic dysfunction-associated fatty liver disease” OR “metabolic-associated fatty liver disease” OR “metabolism-related fatty liver” OR NASH OR “nonalcoholic steatohepatitis” OR “nonalcoholic steatohepatitides” OR “non-alcoholic steatohepatitis” OR MASH OR “metabolic dysfunction-associated steatohepatitis”) AND TS = (TCM OR “Traditional Chinese Medicine” OR “Chinese Traditional Medicine” OR “Chinese medicine*” OR “Chinese herb*” OR “Chinese drug*” OR “Chinese medicinal herb” OR “Chinese medicinal plant*” OR “Chinese materia medica” OR “Chinese patent medicine*” OR “Chinese medicine compounds” OR “Chinese plant extracts” OR “oral liquid” OR decoction OR formula OR prescription OR tang OR san OR pill OR pellet OR plaster OR capsules OR powder OR granules OR gels) AND DT = (Article OR Review) AND LA = (English)
Scopus	TITLE-ABS-KEY(NAFLD OR “non alcoholic fatty liver disease” OR “nonalcoholic fatty liver disease” OR “non-alcoholic fatty liver disease” OR “nonalcoholic fatty liver” OR MASLD OR “metabolic dysfunction-associated fatty liver disease” OR “metabolic-associated fatty liver disease” OR “metabolism-related fatty liver” OR NASH OR “nonalcoholic steatohepatitis” OR “nonalcoholic steatohepatitides” OR “non-alcoholic steatohepatitis” OR MASH OR “metabolic dysfunction-associated steatohepatitis”) AND TITLE-ABS-KEY(TCM OR “Traditional Chinese Medicine” OR “Chinese Traditional Medicine” OR “Chinese medicine*” OR “Chinese herb*” OR “Chinese drug*” OR “Chinese medicinal herb” OR “Chinese medicinal plant*” OR “Chinese materia medica” OR “Chinese patent medicine*” OR “Chinese medicine compounds” OR “Chinese plant extracts” OR “oral liquid” OR decoction OR formula OR prescription OR tang OR san OR pill OR pellet OR plaster OR capsules OR powder OR granules OR gels) AND DT = (Article OR Review) AND LA = (English)

Search on 31 July 2025. Retrieval time range: 2000−2025.

### 2.2 Data analysis

The yearly publication trends were conducted using Origin 2025. Data visualization and the construction of knowledge maps were carried out through R software (version 4.5.1) integrated with the Bibliometrix package (version 5.0), as well as VOSviewer (version 1.6.20), and CiteSpace (version 6.4. R1) (19–21). To ensure the accuracy of the data and the reliability of the analysis, data extraction and interpretation were performed independently by two authors. The Bibliometrix software was mainly used to analyze annual production, country-wise production, source impact measured by the H-index, and hot topics. VOSviewer was utilized to construct collaboration networks among countries/regions and institutions, conduct co-citation analysis of source, and perform keyword co-occurrence analysis. In co-author network analysis, the parameter settings were as follows: the minimum number of publications of a country/region or an organization ≥5. In the co-citation analysis, the parameter was set to the minimum number of citations of a source ≥20. In the co-occurrence analysis of keywords, the parameters were set as follows: for keywords retrieved from the WoSCC database, the occurrence count ≥10, for keywords retrieved from the Scopus database, the occurrence count ≥50. Moreover, keywords such as “TCM,” “NAFLD,” and their synonyms were excluded. CiteSpace was utilized to detect citation bursts in the reference list and draw the timeline chart of keywords. Specifically, the time span ranged from January 2000 to July 2025, with each slice representing a 1-year interval. The selection criterion was the g-index (*k* = 25). For the setting of network pruning functional regions, the Pathfinder algorithm was chosen in this study, and the Pruning networks method was used as an auxiliary pruning strategy, while other parameters were maintained at their default values. The Kleinberg’s burst detection algorithm was applied to identify emerging trends and sudden changes in citations. Journal Impact Factor (IF) data were retrieved from the 2024 Journal Citation Reports (JCR) to evaluating the academic influence of the journals.

## 3 Results

### 3.1 Trend analysis of annual publication

**Figure 1:**
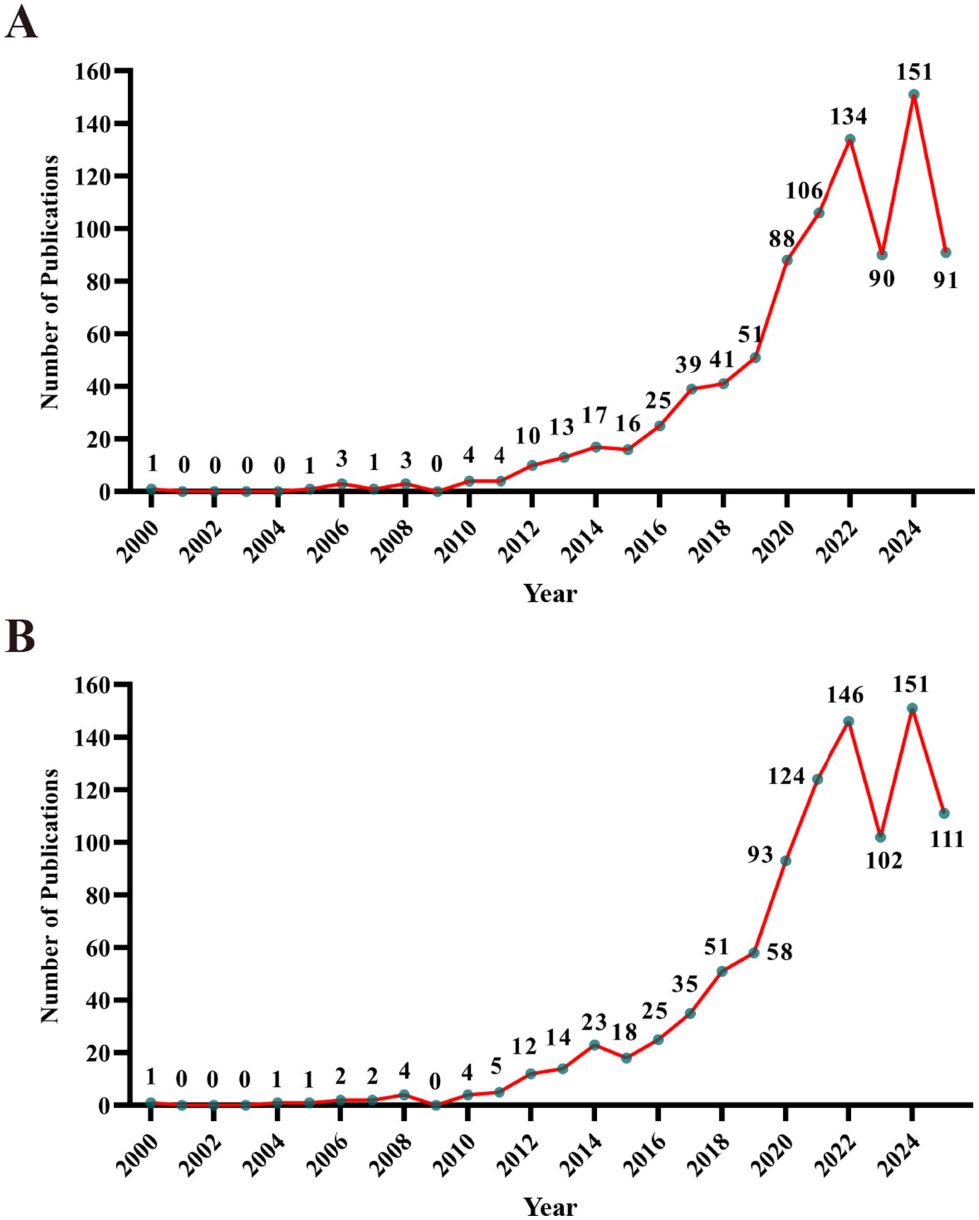
Annual trends in publication outputs on TCM for the treatment of MASLD in **(A)** WoSCC and **(B)** Scopus database.

The annual number of publications serves as an indicator for assessing research dynamics and identifying prevailing trends within a specific field. According to the established retrieval strategy, 889 and 983 articles on TCM treatment for MASLD were retrieved from WoSCC and Scopus databases, respectively. As shown in Figures 1A,B, the annual publication trends in both databases exhibit similar fluctuation patterns. From 2000 to 2025, the annual publication volumes in this field displayed slight fluctuations but maintained an overall upward trend, indicating steadily growing research interest in exploring TCM treatments for MASLD. Notably, since 2019, there has been a dramatic rise in the number of publications, likely driven by the growing global demand for medical resources and alternative therapies during the COVID-19 pandemic. Furthermore, the redefinition of NAFLD as MASLD may have also reinvigorated academic and clinical interest in the disease. Concurrently, the sustained increasing global prevalence of MASLD, accumulating evidence supporting the metabolic benefits of TCM, governmental policy support for TCM development, and the internationalization of TCM-related research have collectively promoted scholarly output in this area.

### 3.2 Contribution of publication output and collaboration in countries/regions and institutions

**Figure 2:**
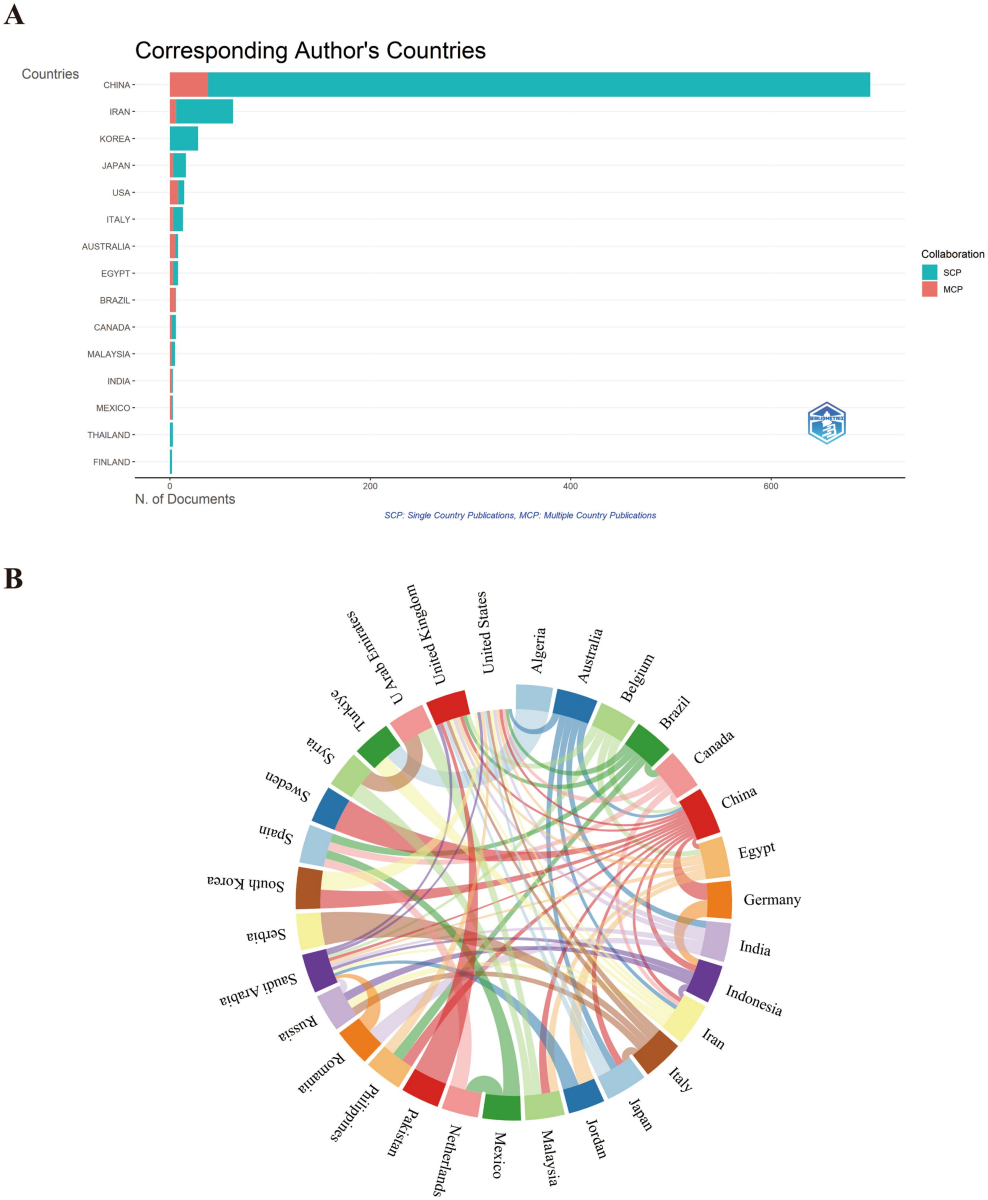
Global trends and contributions of countries/regions to research on TCM in the treatment of MASLD from 2000 to 2025. **(A)** Geographic distribution of corresponding authors and international collaboration. **(B)** Map of cooperation between different countries/regions.

Over the past two decades, a total of 889 articles on TCM treatment for MASLD were published in the WoSCC database. These articles were contributed by corresponding authors from 25 countries/regions. Table 2 presents an overview of the publication distribution, encompassing both single-country and multiple-country contributions. An analysis of the countries associated with corresponding authors indicated that China was the primary contributor (*n* = 699, 78.6%), followed by Iran (*n* = 63, 7.1%), South Korea (*n* = 28, 3.1%), Japan (*n* = 16, 1.8%), and the United States (*n* = 14, 1.6%). Among the top five countries/regions ranked by publication output, China and South Korea exhibited relatively low levels of cross-border publication proportions (MCPs), at 5.4% and 0, respectively, indicating that their research focus remains concentrated domestically. In contrast, the United States and Japan exhibit a greater tendency for international collaboration and global academic exchange, with MCP values reaching 57.1 and 18.8%, respectively. The 983 publications indexed in the Scopus database originated from 28 corresponding author’s countries/regions. Authors from China played a dominant role, publishing 751 articles, which accounted for 76.40% of the total output. Notably, the top five contributing countries in Scopus database coincide with those in the WoSCC database, as shown in Supplementary Table S1. Additionally, to gain further insight into international collaboration patterns, we conducted an analysis of all authors of the papers. Figure 2B presents the worldwide collaborative network among countries indexed in the WoSCC database. The most common collaborative partnership was between China and the United States (17 times), followed by collaborations between Australia and China (13 times), Canada and China (6 times), and China and Japan (5 times). These data indicate that China holds a central position in research on TCM treatment for MASLD and has established collaborative relationships with multiple countries. Utilizing advanced research technologies and methodologies from abroad not only promotes the application of TCM in MASLD treatment but also contributes to advancing the modernization and internationalization of TCM.

**Table 2:** Top 15 countries by productive corresponding authors, scientific impact, and international collaboration in research on TCM treatment of MASLD (WoSCC).

Country (*n* = 25)	Articles	SCP	MCP	Freq	MCP_Ratio
China	699	661	38	0.786	0.054
Iran	63	57	6	0.071	0.095
South Korea	28	28	0	0.031	0
Japan	16	13	3	0.018	0.188
the United States	14	6	8	0.016	0.571
Italy	13	10	3	0.015	0.231
Australia	8	3	5	0.009	0.625
Egypt	8	5	3	0.009	0.375
Brazil	6	1	5	0.007	0.833
Canada	6	4	2	0.007	0.333
Malaysia	5	3	2	0.006	0.4
India	3	1	2	0.003	0.667
Mexico	3	1	2	0.003	0.667
Thailand	3	3	0	0.003	0
Finland	2	2	0	0.002	0

SCP, Single Country Publications; MCP, Multiple Country Publications; MCP_Ratio = MCP/Articles.

**Figure 3:**
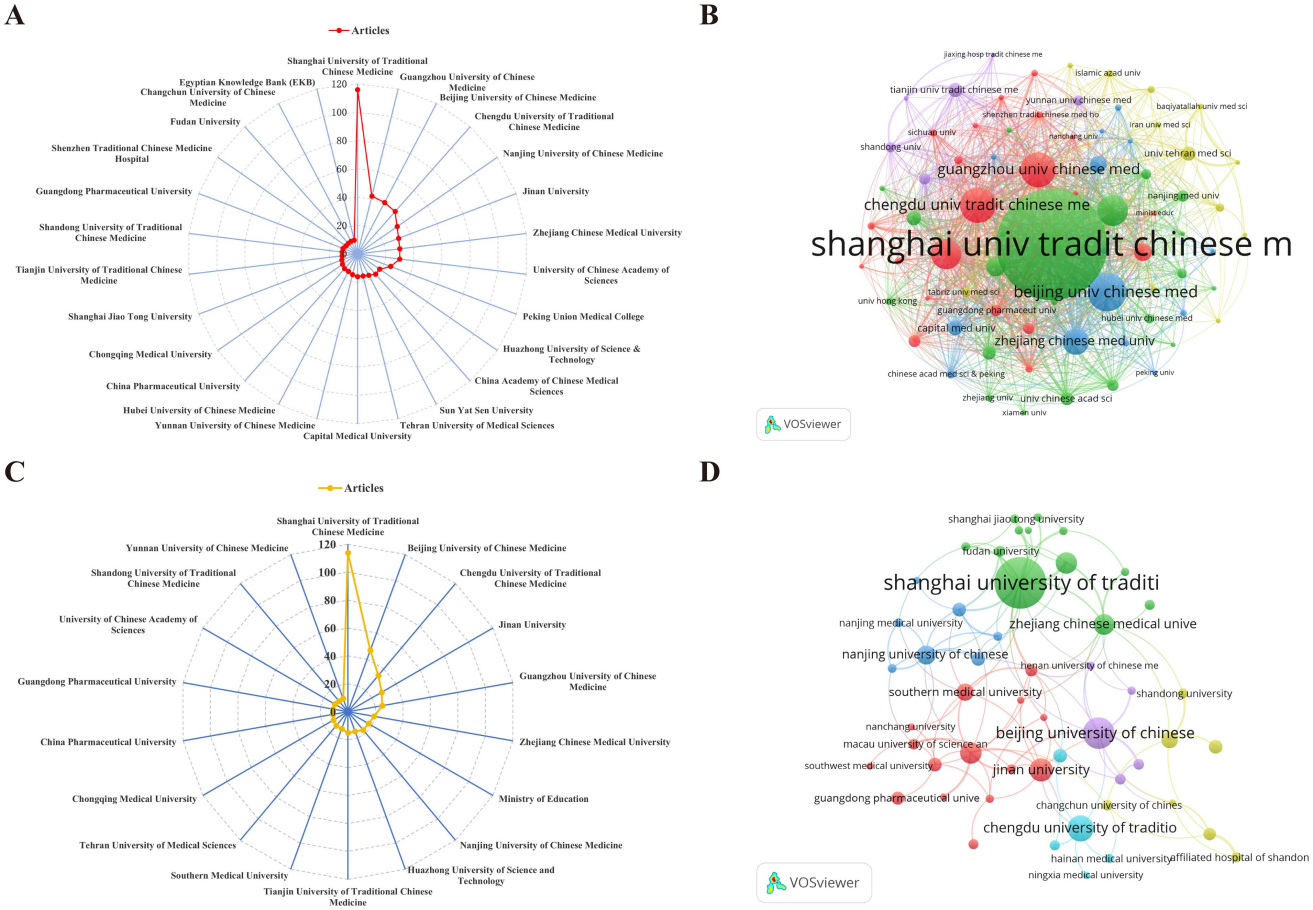
Institutions and their collaborative networks in the field of TCM in MASLD treatment. **(A)** The top 26 institutions with at least 10 publications on TCM in MASLD treatment in the WoSCC database. **(B)** Institutions collaboration analysis in the WoSCC database. **(C)** The top 18 institutions with at least 10 publications on TCM in MASLD treatment in the Scopus database. **(D)** Institutions collaboration analysis in the Scopus database.

Statistical analysis showed that the top 10 institutions with the highest publications in both the WoSCC and Scopus databases were major Chinese medicine universities in China (Figures 3A,C). Among them, Shanghai University of Traditional Chinese Medicine has made the most significant contributions in this field, publishing 116 papers in the WoSCC database and 114 articles in the Scopus database. Guangzhou University of Chinese Medicine ranked second in WoSCC with 42 publications and fifth in Scopus with 25 publications. These institutions acted as the core driving force behind TCM research, offering strong academic support for the inheritance and innovative development of TCM.

Additionally, a collaborative network analysis was performed for institutions that published more than five papers, as illustrated in Figures 3B,D. The findings indicate that collaborative relationships have been established among various institutions, with higher-publishing institutions demonstrating more extensive cooperative networks with other institutions. These findings suggest that institutions can foster academic exchange and enhance research quality by strengthening collaboration.

### 3.3 Distribution of the most productive and active journals

The distribution of publications in academic journals reflects the landscape of primary communication platforms within the field. The top 10 journals, ranked by the number of publications, h-index, total citations (TC), and Impact factors (IF), are listed in Table 3 for both the WoSCC and Scopus databases. *Journal of Ethnopharmacology* ranked first in both databases, with 88 articles (9.91%) in WoSCC and 93 articles (9.46%) in Scopus. Furthermore, it also exhibited the highest h-index (WoSCC: 21, Scopus: 23) and the greatest number of total citations (WoSCC: 1,230, Scopus: 1,434), reflecting its central role and extensive academic influence in this research area. In terms of publication volume, the next most productive journals are *Frontiers in Pharmacology* (WoSCC: 74, Scopus: 69) and *Evidence-Based Complementary and Alternative Medicine* (WoSCC: 56, Scopus: 54). Among the top 10 journals ranked by the number of publications, *Phytomedicine* boasts the highest impact factor (IF = 8.3). Notably, the relatively limited number of studies on TCM for MASLD treatment published in high-impact journals highlights the need to enhance research quality in this domain.

**Table 3:** The top 10 most productive and active journals published research on the treatment of MASLD with TCM.

Source, WoSCC	H-index	TC	NP	(%)	IF (2025)
Journal of Ethnopharmacology	21	1230	88	9.91	5.4
Frontiers in Pharmacology	19	1069	74	8.33	4.8
Evidence-based Complementary and Alternative Medicine	15	671	56	6.31	—
Phytomedicine	12	410	33	3.72	8.3
Biomedicine and Pharmacotherapy	16	810	30	3.38	7.5
Nutrients	9	207	14	1.58	5
World Journal of Gastroenterology	8	360	12	1.35	5.4
Chinese Medicine	7	186	11	1.24	5.7
Phytotherapy Research	7	235	11	1.24	6.3
Journal of Traditional Chinese Medicine	4	61	11	1.24	2.2
Source, Scopus					
Journal of Ethnopharmacology	23	1434	93	9.46	5.4
Frontiers in Pharmacology	20	1106	69	7.02	4.8
Evidence-based Complementary and Alternative Medicine	16	730	54	5.49	—
Phytomedicine	14	537	38	3.87	8.3
Biomedicine and Pharmacotherapy	17	836	34	3.46	7.5
Nutrients	12	309	17	1.73	5
World Journal of Gastroenterology	10	471	17	1.73	5.4
Phytotherapy Research	8	285	14	1.42	6.3
International Journal of Molecular Sciences	10	494	13	1.32	4.9
BMC Complementary Medicine and Therapies	7	118	11	1.12	3.4

H-index, Hirsch index; TC, total citations; NP, number of publications; IF, impact factors.

### 3.4 Citation bursts

**Figure 4:**
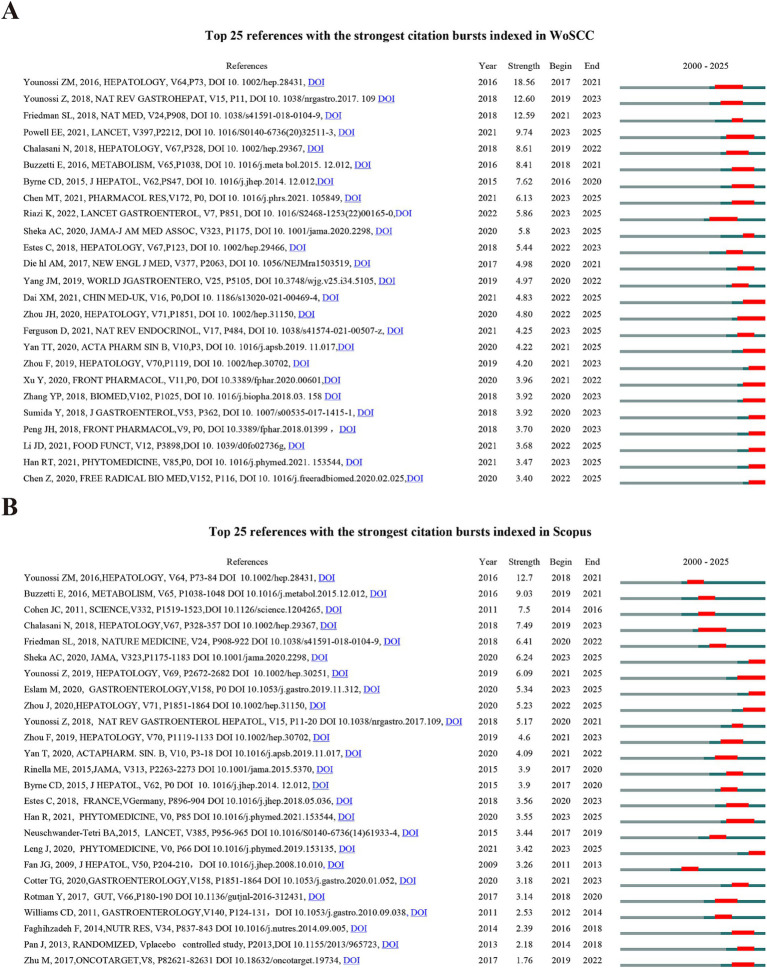
Top 25 references with the strongest citation bursts on TCM for MASLD treatment research based on data from the **(A)** WoSCC and **(B)** Scopus databases.

To further explore the knowledge base and developmental frontiers in TCM for the management of MASLD, this study employed CiteSpace to detect the 25 most prominent citation bursts in this domain (refer to Figure 4). The corresponding titles and DOIs are provided in Supplementary material 2. The three publications with the highest citation bursts strength in the WoSCC database were: (1) ‘‘Global epidemiology of nonalcoholic fatty liver disease-Meta-analytic assessment of prevalence, incidence, and outcomes” (strength:18.56), (2) ‘‘Global burden of NAFLD and NASH: trends, predictions, risk factors and prevention” (strength:12.6), (3) ‘‘Mechanisms of NAFLD development and therapeutic strategies” (strength:12.59). Further analysis indicates that the citation frequencies of the following three papers have increased significantly in recent years: (1) ‘‘Traditional Chinese Medicine in the treatment of nonalcoholic steatohepatitis,” (2) ‘‘Herbal drug discovery for the treatment of nonalcoholic fatty liver disease,” (3) ‘‘Non-alcoholic fatty liver disease” (Figure 4A).

The three publications with the highest citation bursts strength in the Scopus database were: (1) ‘‘Global epidemiology of nonalcoholic fatty liver disease-Meta-analytic assessment of prevalence, incidence, and outcomes” (strength:12.7), (2) ‘‘The multiple-hit pathogenesis of non-alcoholic fatty liver disease (NAFLD)” (strength:9.03), (3) ‘‘Human fatty liver disease: old questions and new insights” (strength:7.5). Additionally, in recent years, the citation counts of the following three studies have increased significantly: (1) ‘‘The global epidemiology of nonalcoholic fatty liver disease (NAFLD) and nonalcoholic steatohepatitis (NASH): a systematic review,” (2) ‘‘MASLD: A Consensus-Driven Proposed Nomenclature for Metabolic Associated Fatty Liver Disease,” (3) ‘‘Si Miao Formula attenuates non-alcoholic fatty liver disease by modulating hepatic lipid metabolism and gut microbiota” (Figure 4B).

The temporal distribution and intensity variations of reference citations visually reveal the evolution of research hotspots and changes in the knowledge base within the field of TCM treatment for MASLD. Through citation burst analysis, three core research directions in the area of TCM for MASLD treatment were identified: 1. Investigating the therapeutic targets and underlying mechanisms of TCM interventions, with a focus on key biological pathways associated with lipid metabolism, insulin resistance, oxidative stress, and gut microbiota regulation. 2. Modernization of TCM and the development of new drugs through methods such as network pharmacology, reverse pharmacology, and reverse pharmacokinetics. 3. Clinical research on TCM for MASLD treatment aims to establish high-level evidence-based medicine by comprehensively assessment of its effectiveness and safety.

### 3.5 Keyword co-occurrence and evolutionary trends

**Figure 5:**
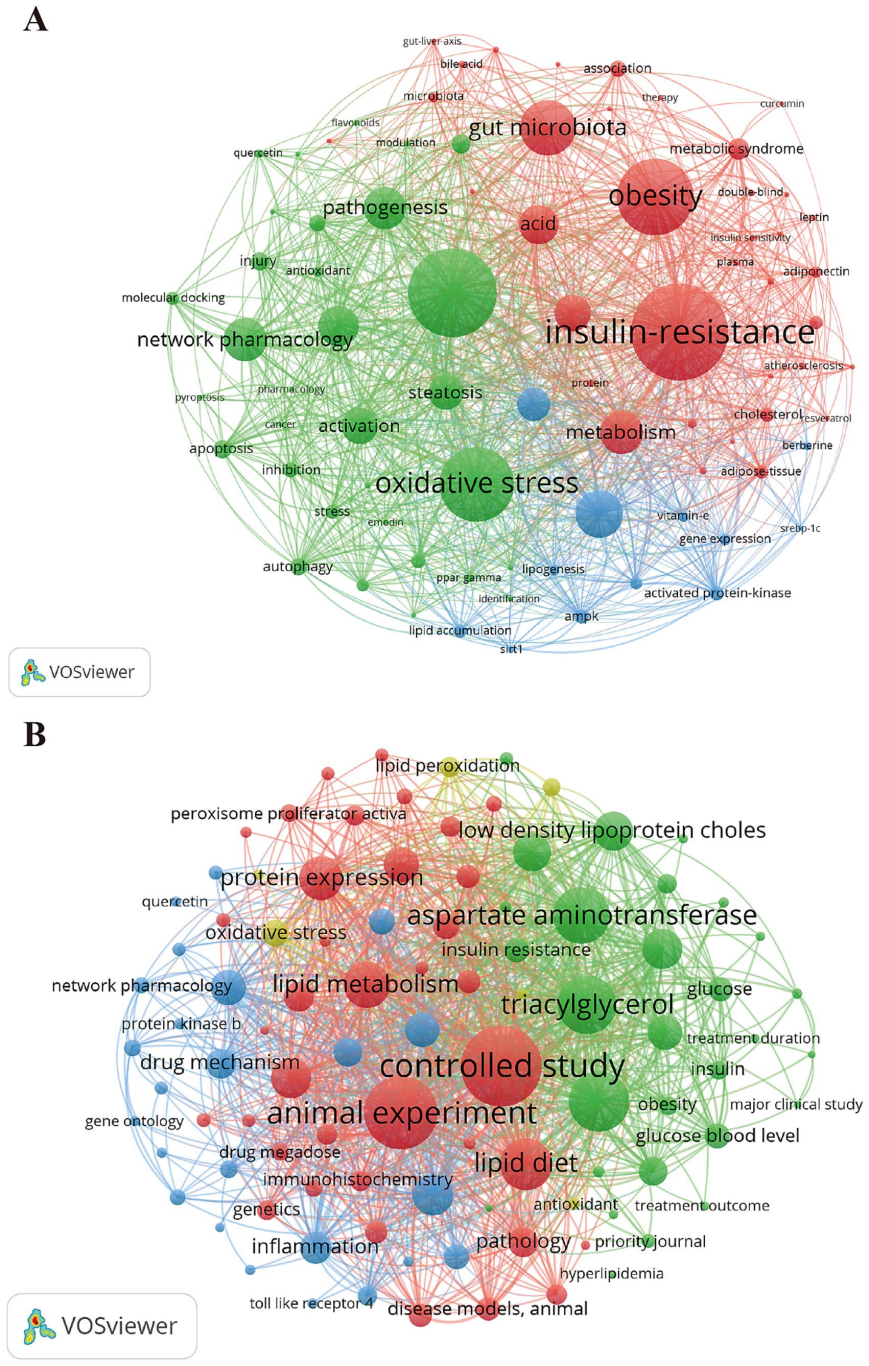
The visualization map of keyword co-occurrence on TCM for MASLD treatment research based on data from the **(A)** WoSCC and **(B)** Scopus databases.

Keywords provide a concise summary of the document’s content. Analyzing keywords helps to identify research hotspots and cutting-edge directions in a certain field. In our study, 487 keywords were identified in WoSCC and 783 keywords in Scopus using VOSviewer. Through cluster analysis, keywords in WoSCC were observed to form three different colored clusters. (1) The effects of TCM on MASLD and related metabolic syndrome (red dot) involves a total of 38 keywords, such as insulin resistance, obesity, gut microbiota, metabolic syndrome, adiponectin, hyperlipidemia, atherosclerosis, leptin, diabetes and so on. (2) The molecular mechanism of TCM in treating MASLD (green dot) encompass 32 keywords, such as inflammation, oxidative stress, apoptosis, autophagy, endoplasmic reticulum stress, network pharmacology, molecular docking, and so on. (3) The regulation of lipid metabolism in MASLD by TCM and its associated targets (blue dot) involves 12 keywords, including lipid metabolism, mechanism, lipid accumulation, peroxisome proliferator247 activated receptor *α* (PPARα), lipogenesis, BBR, SIRT1, sterol regulatory element-binding protein- 1c (SREBP-1c), and so on (Figure 5A). A complete list of keywords within all three clusters is provided in Supplementary material 3.

Figure 5B displays the keyword co-occurrence network in the Scopus database, depicting four major clusters. (1) Mechanisms and research methods of TCM in treating MASLD (red dot), with 37 keywords, including controlled study, animal experiment, lipid metabolism, pathogenesis, gut microbiota, drug dosage, ultra performance liquid chromatography, metabolomics and so on. (2) Clinical efficacy assessment of TCM treatment for MASLD (green dots), with 29 keywords including alanine aminotransferase (ALT), aspartate aminotransferase (AST), triacylglycerol (TG), cholesterol (TC), low density lipoprotein cholesterol (LDL-c), high density lipoprotein cholesterol (HDL-c), insulin resistance, treatment duration, treatment outcome and so on. (3) Pharmacological mechanisms and targets of TCM anti-inflammatory effects (blue dots), with 24 keywords including inflammation, tumor necrosis factor *α* (TNF-α), interleukin-6 (IL-6), interleukin-1β (IL-1β), apoptosis, network pharmacology, peroxisome proliferator-activated receptor *γ* (PPARγ), Toll-like receptor 4 (TLR4), molecular docking, bioinformatics and so on. (4) Anti-oxidant effects of TCM (yellow dots), with 6 keywords including oxidative stress, antioxidant, superoxide dismutase, lipid peroxidation and so on.

**Figure 6:**
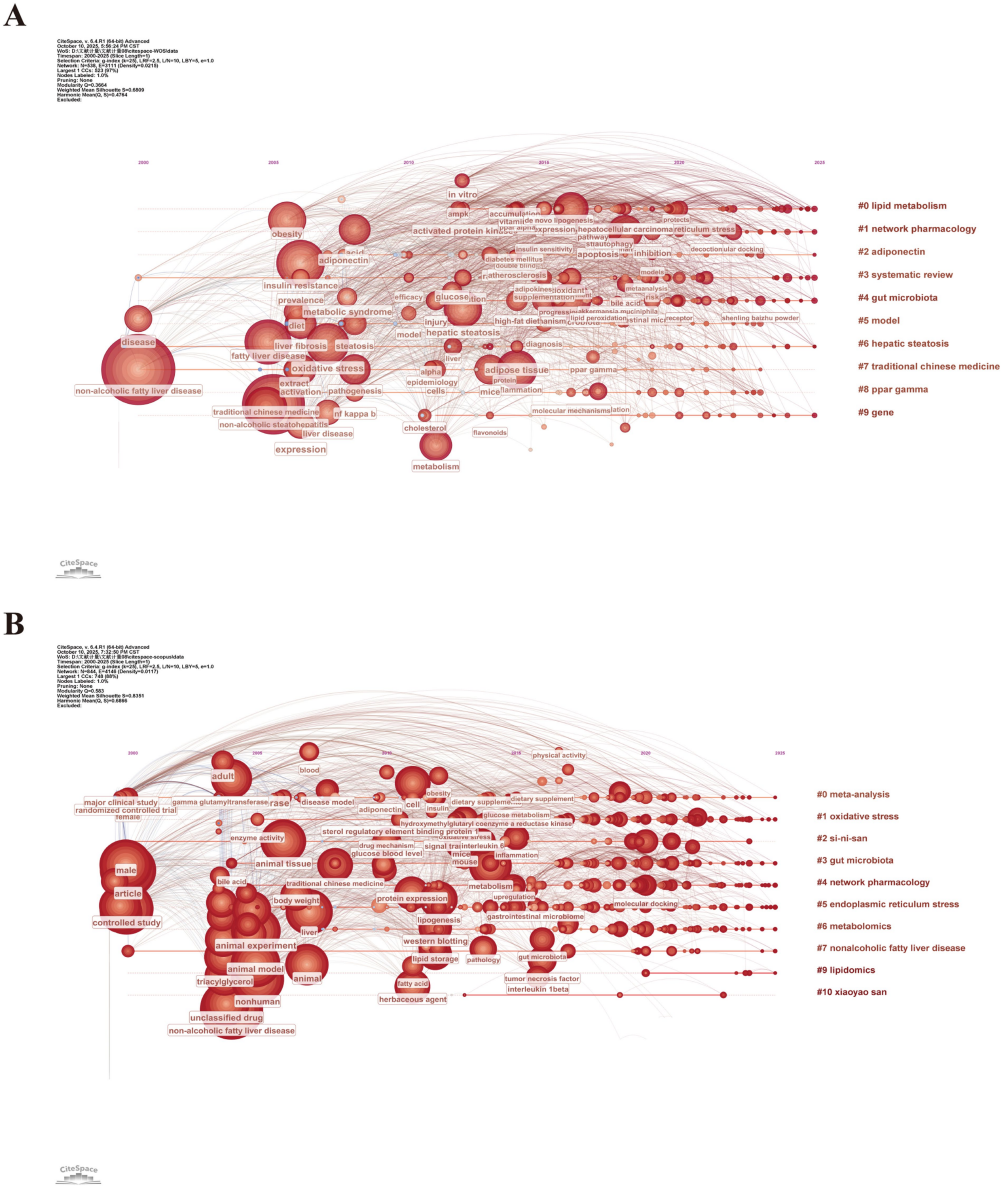
Timeline of keywords on TCM for MASLD treatment research based on data from the **(A)** WoSCC and **(B)** Scopus databases.

A timeline diagram can clearly illustrate the timeframe scope of each research focus and highlight the connections between different research hotspots, thereby systematically presenting the evolution of research priorities in TCM treatment for MASLD and offering insights into future research directions. Figure 6A shows the cluster of the keywords in conjunction with the time from 2000 to 2025. From 2000 to 2010, keywords such as “NAFLD,” “Traditional Chinese Medicine,” “obesity,” “insulin resistance,” “liver fibrosis,” “metabolic syndrome,” and “adiponectin” attracted more attention. This marked the initial phase of research primarily focused on descriptive studies and preliminary exploration of mechanisms. For instance, researchers explored the effects of TCM on clinical symptoms, blood biochemistry, and liver imaging characteristics in MASLD patients. From 2011 to 2020, as the understanding of MASLD pathogenesis deepened, research on TCM treatment for MASLD gradually shifted toward investigating its underlying molecular mechanisms. Emerging keywords included “AMPK,” “de novo lipid synthesis,” “apoptosis,” “autophagy,” “inflammation,” and “network pharmacology.” Researchers utilized multi-omics analysis and network pharmacology to elucidate the multi-component, multi-target characteristics of TCM, reflecting a transition from descriptive studies to mechanism-oriented investigations. Since 2021, with a deepening understanding of the gut-liver axis and the emergence of methods such as molecular docking, keywords within clusters “#1 Network Pharmacology” and “#4 Gut Microbiota” have gained significant attention. This trend indicates that precision intervention strategies targeting gut microbiota and specific molecular targets have emerged as new research directions.

Figure 6B shows the timeline graph of keywords in the Scopus database. In the early stages, high-frequency keywords included “controlled study,” “animal model,” “animal experiment,” “animal tissue,” “*γ*-glutamyl transferase,” and “TG,” reflecting that research primarily focused on evaluating the overall efficacy of TCM through randomized controlled trials and animal experiments. From 2010 to 2020, research focus gradually shifted toward molecular mechanisms. Key terms during this period included “protein expression,” “metabolism,” “inflammation,” “lipid storage,” “gut microbiota,” and “pathology.” In recent years, keywords within clusters “#1 oxidative stress,” “#2 Sini San,” “#3 gut microbiota,” “#4 network pharmacology,” and “#6 metabolomics” have formed the current research frontiers. The timeline analysis indicates that future research will integrate multi-omics technologies to explore the molecular mechanisms of TCM treatment for MASLD in greater depth. Furthermore, obtaining more comprehensive and reliable clinical evidence will be essential for promoting the in-depth development of TCM research in the context of MASLD.

### 3.6 Comprehensive analysis of hotspots

In summary, through a comprehensive analysis, including citation bursts, keyword clustering, and timeline diagrams, this study has identified emerging trends and cutting-edge areas in the application of TCM for MASLD treatment (Supplementary Figure S1). The results suggest that current research focus in this area primarily centers on three core themes: (1) Elucidating the mechanisms of TCM intervention in MASLD. (2) Clinical research and practical applications of TCM in MASLD treatment. (3) Discovery and application of bioactive compounds derived from TCM for MASLD treatment.

## 4 Discussion

### 4.1 General information

This research performed a systematic review of literature related to TCM and MASLD published in the WoSCC and Scopus databases from 2000 to 2025. The findings indicate that the annual number of publications on TCM treatment for MASLD has shown a consistent upward trend, reflecting an increasing research interest in the application of TCM for the treatment of MASLD. China ranks first in publication output, with the 10 most productive institutions all located within the country. This may be attributed to the long history of TCM application in China and sustained governmental support for its development (22). Recently, the Chinese government has substantially increased funding for modernizing TCM and promoting its integration with Western medicine practices, particularly through national initiatives like the “Healthy China 2030” strategy and the inclusion of TCM into official guidelines for the prevention and treatment of chronic disease (23). Significant investment in research, specialized institutions focused on TCM, and favorable regulatory policies have enabled Chinese organizations to lead and conduct large-scale, multidisciplinary studies. As a result, China has become an international center for research in this field. However, although Chinese institutions account for the largest proportion of research outputs, these achievements predominantly originate from economically developed regions such as Shanghai, Guangzhou, and Beijing. This uneven geographical distribution may hinder regional exchange and collaboration. The *Journal of Ethnopharmacology* emerges as the most influential publication in this domain, with the highest publication volume and citation count, making it the leading platform for disseminating TCM research.

### 4.2 Hotspots and development trends

Through bibliometric analysis including literature clustering, keyword co-occurrence, and topic evolution, this study identifies the key research hotspots and developmental trends in the field of TCM treatment for MASLD. The core research domains encompass the following three aspects:

#### 4.2.1 Mechanism analysis of TCM intervention in MASLD

Based on the principles of “holistic view” and “dialectical treatment,” TCM has demonstrated significant effectiveness in the prevention and management of MASLD (12). In recent years, the application of multi-omics approaches—such as transcriptomics, metabolomics, proteomics, and microbiomics—has deepened the understanding of TCM’s mechanisms. Multiple studies suggest that TCM exerts hepatoprotective effects through diverse pathways, such as regulating lipid metabolism, reducing inflammation and fibrosis, mitigating oxidative stress, and modulating gut microbiota composition. As a result, TCM has become an increasingly crucial strategy for addressing the complex pathophysiology of MASLD (24).

##### 4.2.1.1 Regulating lipid metabolism and energy homeostasis

Analysis of keywords in this study indicates that “regulating lipid metabolism” is one of the core themes in research on the mechanisms of TCM treatment for MASLD. The blue cluster in Figure 5A and its rise as a focus during 2011–2020 in Figure 6A both indicate that lipid metabolism regulation has received extensive attention in this field. Hepatic lipid dysregulation represents the initial step in the development of MASLD (25). The liver accumulates lipids through two primary pathways: the uptake of circulating free fatty acids and *de novo* lipogenesis. Conversely, lipids are eliminated via *β*-oxidation and secretion as very-low-density lipoprotein (VLDL) (26). Extensive research indicates that TCM can effectively ameliorate MASLD by regulating lipid synthesis and metabolic pathways. For example, lipidomics analysis demonstrated that Compound Shouwu Jiangzhi Granule modulates hepatic diacylglycerols metabolism in MASLD mice. This intervention led to a decrease in the levels of MASLD-inducing glycerides, such as diacylglycerols and triacylglycerols. Meanwhile, it caused an increase in the levels of MASLD-protective phospholipids, including phosphatidic acids, phosphatidylethanolamines, and phosphatidylglycerols (27). Through integrated metabolomics and transcriptomics analyses, Cao et al. (28) demonstrated that the Zexie-Baizhu Decoction regulates the expression of proteins associated with lipid biosynthesis, catabolism, and transport by activating two energy-sensing molecules, SIRT1 and AMPK. This activation leads to the suppression of lipogenesis and enhancement of fatty acid oxidation, effectively improving lipid metabolism in mouse models of MASLD. Furthermore, a study integrating metabolomics with molecular docking and experimental validation revealed that the combined use of *Scutellariae Radix* and *Coptidis Rhizoma* exhibits synergistic hypolipidemic effects in both rat models and HepG2 cells. Specifically, *Scutellariae Radix* enhances SIRT6-mediated deacetylation, upregulates acyl-CoA synthetase long-chain proteins member 5 (ACSL5) activity, and enhances fatty acid oxidation. Meanwhile, *Coptidis Rhizoma* exerts its effect by inhibiting stearoyl-CoA desaturase 1 (SCD1), a key enzyme in lipid biosynthesis. Their combined action effectively restores hepatic lipid homeostasis (29).

##### 4.2.1.2 Inhibiting liver inflammatory response

Through cluster analysis, we found terms such as “inflammation,” “TNF-α,” and “IL-6” formed an independent cluster. This finding indicates that the inflammatory response has emerged as one of the core research directions in TCM treatment for MASLD. Hepatic inflammatory infiltration plays a crucial part in driving the progression of MASLD to MASH (30). Pro-inflammatory cytokines, such as TNF-*α* and IL-6, not only exacerbate hepatic lipid accumulation but also stimulate collagen synthesis by activating hepatic stellate cells (HSCs), thus triggering the onset of liver fibrosis (31). Research has found that TCM can interrupt this pathological cascade by modulating crucial signaling nodes, thereby exerting anti-inflammatory effects in the treatment of MASLD. For example, Gegen Qinlian Decoction and Wang’s empirical formula have shown efficacy in mitigating hepatic inflammation in MASH mice by inhibiting the activation of the TLR4 signaling pathway, thereby suppressing the secretion of pro-inflammatory cytokines (32, 33). Nine hundred nineteen syrup modulates the balance between PPARγ/NF-κB (anti-inflammatory) and TNF-α/NF-κB (pro-inflammatory) signaling pathways, thereby suppressing the expression of inflammatory cytokine and improving hepatic pathological damage in MASLD mice (34). Additionally, growing evidence indicates that the activation of NOD-like receptor protein 3 (NLRP3) inflammasomes leads to hepatic lipid deposition, liver injury, and pyroptosis, thereby significantly driving the progression of MASLD (35). Consequently, inhibiting NLRP3 inflammasome is regarded as a potential therapeutic strategy for MASLD. Based on this mechanism, kinsenoside, asperuloside, and mangiferin have demonstrated therapeutic effects by suppressing the formation and activation of the NLRP3 inflammasome, thereby mitigating inflammatory responses and pyroptosis (36–38).

##### 4.2.1.3 Inhibiting liver fibrosis

In keyword co-occurrence analysis, “liver fibrosis” emerged as a high-frequency term in the field of TCM treatment for MASLD, and it has received sustained attention over time. This finding suggests that anti-fibrotic effects represent a consistently significant research direction in TCM treatment for MASLD. Liver fibrosis is a severe pathological process during the development of MASH, marked by HSCs activation and the abnormal buildup of extracellular matrix (39). In healthy livers, HSCs remain quiescent; however, under conditions of chronic liver injury, HSCs activate into *α*-smooth muscle actin (α-SMA)-expressing myofibroblasts, which drive the proliferation of fibrous tissue (40). Accumulating evidence suggests that multiple bioactive constituents derived from TCM exhibit anti-fibrotic potential. Treatment with ginsenoside Rg5, gypenosides, tanshinone IIA, and betaine has been shown to significantly suppress the expression of fibrosis-associated markers, including α-SMA, Collagen I, and transforming growth factor-β (TGF-β) in the liver of mice with MASH (41–44). Proteomics data indicated that triptolide is a potential allosteric AMPK agonist, shown to ameliorate hepatic lipogenesis and fibrosis in both db/db mice and mice fed a methionine/choline-deficient (MCD) diet through AMPK activation (45).

##### 4.2.1.4 Modulating gut microbiota

In recent years, the role of “gut microbiota” in the treatment of MASLD with TCM has attracted significant attention. Both the results of keyword clustering and timeline view suggest that “gut microbiota” represents an emerging frontier in this field. Dysbiosis of the gut microbiota is a hallmark features of MASLD, and restoration of microbial homeostasis may contribute to the ameliorating this condition (46). TCM exerts therapeutic effects through the regulation of gut microbiota. For instance, Shenling Baizhu San was able to modulate the gut microbiota dysbiosis in MASLD mice by significantly enhancing the relative abundance of probiotics and suppressing the proliferation of pro-inflammatory bacteria, thereby reducing hepatic lipid accumulation and inflammatory responses (47). Intervention with Cassiae Semen significantly decreased hepatic lipid buildup in mice fed a HFD, restored intestinal barrier function, and suppressed inflammatory responses. Moreover, the pharmacological effects of Cassiae Semen were successfully transferred to MASLD recipient mice via fecal microbiota transplantation. However, antibiotic-induced gut microbial disruption abolished or significantly diminished its protective effects, highlighting the essential role of gut microbiota in mediating the pharmacological activity of Cassiae Semen (48). Additionally, a prospective, randomized, controlled clinical trial has observed that 12 weeks of oral administration of the spleen-strengthening and liver-draining formula partially restored gut microbiota imbalance in MASLD patients, particularly affecting taxa such as Coprococcus, Lachnospiraceae_NK4A136 group, and Ruminococcus genus (49).

#### 4.2.2 Clinical application and research of TCM in the treatment of MASLD

##### 4.2.2.1 TCM improves core clinical indicators in patients with MASLD

In the keyword co-occurrence network, terms that reflect clinical efficacy and safety, including liver function, blood lipids, “insulin resistance,” and “treatment outcomes,” are highly clustered. This indicates that clinical efficacy assessment is one of the key focal points in TCM research field. Chinese medicine doctors perform a thorough evaluation of patients’ particular symptoms, mental state, tongue appearance, and pulse characteristics to formulate individualized treatment plans, including adjustable medication combinations and treatment durations (12). Clinical studies have confirmed that TCM not only alleviates patients’ systemic symptoms, but also reduces body mass index (BMI), regulates metabolic disorders, improves liver function, and reduces hepatic fat content. A multicenter, randomized, double-blind, double-dummy clinical trial demonstrated that receive Qushi Huayu for 24 weeks significantly improved AST and ALT levels and reduced liver fat content in MASLD patients (50). Furthermore, compared to MASLD patients who received only dietary and lifestyle interventions, those treated with Linghe Granules showed marked reductions in hepatic fat deposition and significant improvements in key indicators, including BMI, liver function markers (AST and ALT), TG, and LDL-c levels (51). Notably, TCM external therapies have also showed potential in the management of MASLD. Clinical evidence suggests that after 2 weeks of herbal decoction colon hydrotherapy, MASLD patients exhibited reductions in BMI, blood lipids (TG, TC), and liver enzymes (ALT, AST, and *γ*-glutamyl transferase) levels (52). Although further high-quality, large-scale clinical trials are needed to confirm the underlying mechanisms and long-term effects of TCM external interventions, these findings provide new insights for diversified treatment strategies for MASLD.

##### 4.2.2.2 Integration of TCM and Western medicine for the treatment of MASLD

Given the complex pathophysiological processes underlying MASLD, single-target drugs have shown limited efficacy. As a result, multi-drug combination therapy strategies have attracted growing interest in clinical research. Western medicine offers rapid symptom amelioration, while TCM focuses on holistic balance and regulation. Integration of TCM and Western medicine for the treatment of MASLD may capitalize their strengths, thereby improving overall treatment outcomes. This integrated therapeutic strategy has become a promising direction for clinical investigation (53). A retrospective study demonstrated that the combining *Dendrobium nobile* powder with conventional therapies for liver protection and enzyme reduction (phosphatidylcholine capsules and bicyclol) not only significantly improved liver function and lipid metabolism in patients, but also reduced serum inflammatory factor levels and ameliorated liver fibrosis indices (54). Additionally, the combined use of Danshao Shugan Granules and silibinin improved B-ultrasonography findings in MASLD patients and reduced their TCM symptom and sign scores (55). It is evident that the integrated use of TCM and Western medicine not only helps improves objective pathological parameters but also helps alleviate patients’ clinical symptoms. Moreover, it partially compensates for the limitations of Western medicine in reversing liver fibrosis and related conditions. However, current evidence is primarily derived from small-sample clinical observations. Large-scale clinical studies are urgently needed to further confirm the effectiveness, thereby providing clinical evidence for the integrated management of MASLD using both Chinese and Western medicine (53).

#### 4.2.3 Research and development of bioactive compounds in TCM for the treatment of MASLD

In keyword co-occurrence analysis, the emergence of terms such as “berberine” and “quercetin” indicates that the identification and functional research of bioactive compounds in TCM have become a significant focus in this field. Due to the complex and unclear composition of TCM, as well as the incomplete understanding of action mechanisms, its widespread clinical application faces significant challenges (24). In recent years, the sustained advancement and integrated application of multidisciplinary approaches such as network pharmacology, omics technologies, and serum pharmacochemistry have facilitated the identification of bioactive compounds in TCM and enhanced the clarification of their underlying mechanisms (56). Notably, network pharmacology enables the rapid identification of core active components in TCM and predicts their molecular targets by constructing “component-target-disease” network (57). Taking Erhuang Quzhi Granules as an example, researchers screened 12 active ingredients—such as Apigenin, Aloe-Emodin, Emodin—by assessing their absorption, distribution, metabolism, and excretion properties. Molecular docking analyses confirmed these components form stable binding structures with heat shock protein 90 alpha family class A member 1 (HSP90AA1), a core target of MASLD. Subsequent *in vivo* experiments further confirmed that these biological compounds improve liver inflammation and lipid deposition in MASLD mice by inhibiting the HSP90/NF-κB/NLRP3 pathway, thereby clarifying the material basis and molecular mechanism of the Erhuang Quzhi Granules (58). Omics technologies can systematically reveal the holistic regulatory effects of TCM, and when combined with network pharmacology, facilitate the reverse prediction of bioactive components. For example, researchers identified core targets regulated by Xiaoyao San through high-throughput sequencing. Subsequently, a “component-target-pathway” network was constructed using network pharmacology analysis. By integrating molecular docking analysis, narcissin, casuarictin, and *γ*-sitosterol were identified as key bioactive constituents in Xiaoyao San, which exert anti-fibrotic effects through stable interactions with critical therapeutic targets (59). Furthermore, serum pharmacochemistry provides critical technical support for identifying pharmacologically active substances that exert directly therapeutic effects *in vivo*. Eight crucial components of ShuGan-QieZhi capsule were identified in mouse serum after oral administration of the capsule using HPLC-MS/MS. Subsequent cellular experiments further demonstrated that these compounds exert significant pharmacological activity in reducing hepatic lipid accumulation and attenuating inflammation responses (60). It is noteworthy that the gut microbiota also plays a role in the metabolism and transformation of active components in TCM (61). Although many TCM ingredients demonstrate low bioavailability, their notable therapeutic effects imply that their pharmacological actions might be mediated by biotransformation processes facilitated carried out by gut microbiota. For instance, ginsenoside Rb1 is metabolized by intestinal microbiota after oral administration, resulting in the formation of compound K, which exhibited higher bioavailability, lower toxicity, and greater pharmacological activity (62). In addition, nitroreductases-producing bacteria (NRs) have been recognized as crucial microorganisms that promote the intestinal absorption of BBR. NRs mediate the conversion of BBR into dihydroberberine, a highly hydrophilic metabolite that enables more efficient absorption into bloodstream. Once absorbed, dihydroberberine is subsequently oxidized back to BBR in systemic circulation. Studies have confirmed that a roughly 10% increase in fecal NRs activity within the gut microbiome can lead to a 65–70% rise in plasma BBR levels, substantially enhancing its oral bioavailability and overall therapeutic efficacy (63, 64). Therefore, identifying these key transformative microbial communities and their metabolites has become a crucial strategy for discovering highly effective bioactive compounds in TCM.

### 4.3 Limitations

This research offers a comprehensive analysis of the current state, hotspots, and emerging trends in TCM applied to MASLD, utilizing data retrieved from the WoSCC and Scopus databases. However, this study also has some limitations. First, the analysis was conducted using only two databases (WoSCC and Scopus). The exclusion of other databases may restrict the comprehensiveness of the findings. Second, we only focused on articles published in English. This language bias may lead to the omission of significant non-English literature and impact the integrity of the research conclusions. Nevertheless, it is noteworthy that English is the most widely used language in international academic communication, indicating that the literature incorporated in this study encompasses the majority of research. Additionally, we assessed the most highly cited articles based on their total citation counts. However, recently published articles may receive fewer citations temporarily due to their publication date (65). Finally, the analytical software utilized in this study (CiteSpace and VOSviewer) may yield slightly different statistical results due to variations in algorithm selection and parameter settings. However, our analysis does not solely depend on the software’s statistics data. Based on the obtained results, we conducted a comprehensive analysis to ensure the reliability of our research conclusions. Although these limitations exist, the findings of this study are still highly reliable. Our research provides valuable insights and references for future investigations in this area. Future studies should further integrate basic experiments with clinical practice to comprehensively evaluate the long-term effects of TCM interventions, explore their potential mechanisms of action, and conduct systematic screening and functional validation of key bioactive components.

## 5 Conclusion

This study offers a systematic evaluation of the current research landscapes and advancements in TCM for the treatment of MASLD. The main findings are outlined below:

- TCM-based interventions for MASLD have attracted significant global attention, especially among researchers in China, Iran, South Korea, Japan, and the United States. Scholars from these countries are actively engaged in this field and have established extensive international collaborations.
- In this research field, the *Journal of Ethnopharmacology* and *Frontiers in Pharmacology* are the most active journals. Notably, the *Journal of Ethnopharmacology* has a significant influence, with the highest number of publications and the greatest citation frequency in the field.
- Mechanistic studies on TCM for the treatment of MASLD are currently focusing on aspects such as “regulating lipid metabolism,” “anti-inflammatory and anti-fibrotic effects,” and “reversing gut dysbiosis.” Among these, gut microbial-mediated biotransformation has emerged as a key area of attention.
- The integration of TCM with holistic regulatory properties and Western medicine with liver-protective and lipid-lowering effects has demonstrated synergistic potential and improvement of patients’ quality of life in clinical practice, becoming an important direction in future clinical practice.

This study expands the research horizon in the field of MASLD treatment, providing valuable perspectives for future scientific exploration and clinical applications. Based on these results, we recommend that future studies further integrate TCM with multi-omics technologies and systems biology approaches to clarify underlying mechanisms and facilitate clinical translation. Furthermore, the organic integration of TCM and Western medicine should be enhanced, along with the advancement of high-quality clinical trials and international, cross-disciplinary cooperation to maximize scientific and therapeutic efficacy. For policymakers, ensuring continuous financial support, establishing robust regulatory systems, and fostering international collaboration are crucial measures to advance the modernization and internationalization of TCM.

## Data Availability

The original contributions presented in the study are included in the article/Supplementary material, further inquiries can be directed to the corresponding author.

## Supplementary material

SUPPLEMENTARY FIGURE 1 A comprehensive analytical framework for identifying research hotspots.
