# Supplementary figures and images for: Mapping the global research output of Traditional Chinese Medicine in the treatment of metabolic dysfunction-associated steatotic liver disease: a comprehensive bibliometric analysis based on multiple databases (2000–2025)
Source: Front Med (Lausanne). 2026 Mar 25;13:1754639. doi: 10.3389/fmed.2026.1754639 (PMC13057568; doi:10.3389/fmed.2026.1754639)

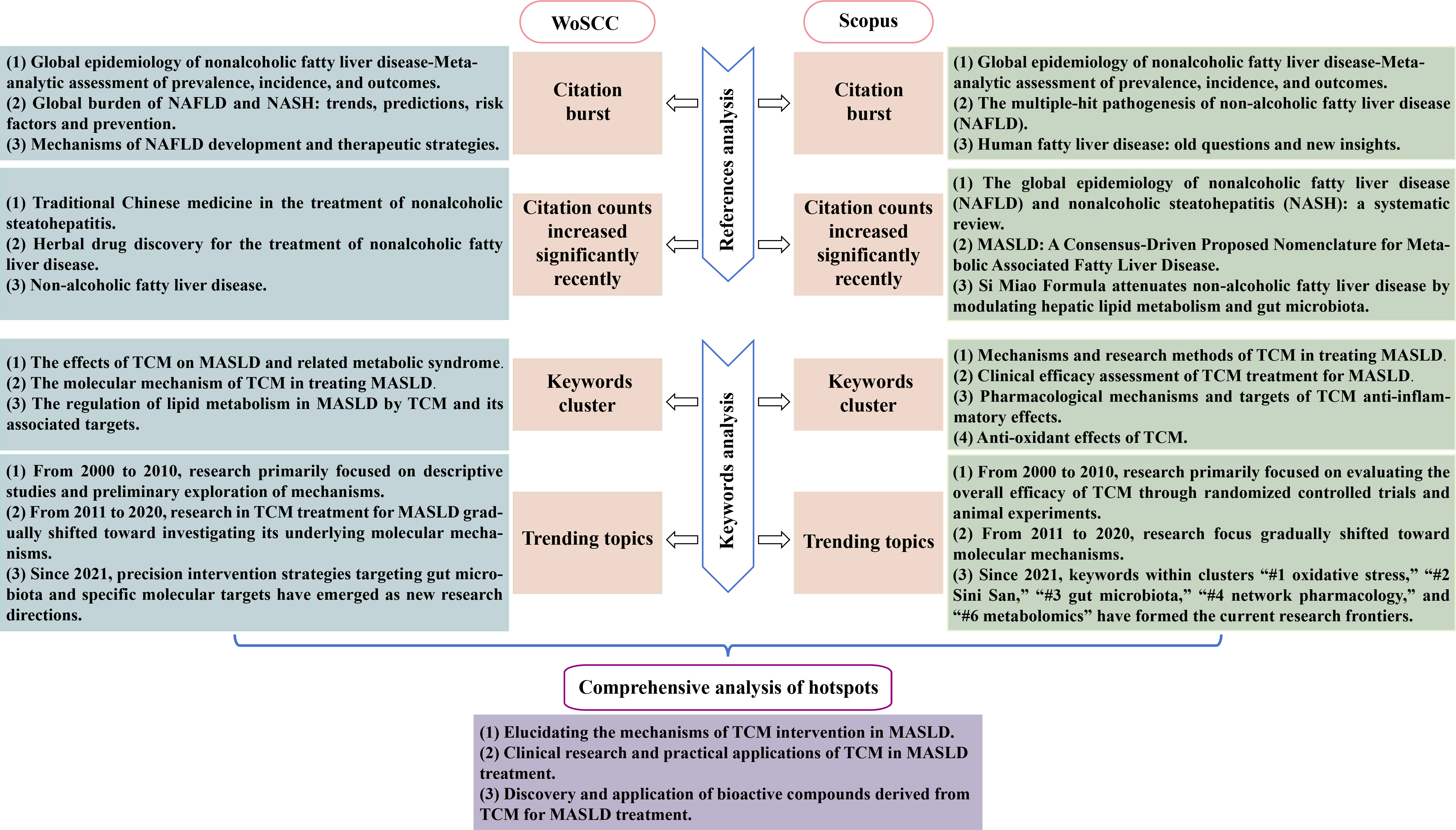

Supplement: SUPPLEMENTARY FIGURE 1 — A comprehensive analytical framework for identifying research hotspots. [file Image_1.tif]
