# Supplementary material for: Mapping the global research output of Traditional Chinese Medicine in the treatment of metabolic dysfunction-associated steatotic liver disease: a comprehensive bibliometric analysis based on multiple databases (2000–2025)
Source: Front Med (Lausanne). 2026 Mar 25;13:1754639. doi: 10.3389/fmed.2026.1754639 (PMC13057568; doi:10.3389/fmed.2026.1754639)
Supplement: Supplementary file 2 [file Table_1.docx]

| **Country（n=28)** | **Articles** | **Freq** |
| --- | --- | --- |
| China | 751 | 0.763 |
| Iran | 66 | 0.067 |
| South Korea | 44 | 0.044 |
| Japan | 22 | 0.022 |
| the United States | 18 | 0.019 |
| Egypt | 8 | 0.008 |
| Italy | 8 | 0.008 |
| Brazil | 6 | 0.006 |
| Australia | 4 | 0.004 |
| Canada | 4 | 0.004 |
| India | 4 | 0.004 |
| Malaysia | 4 | 0.004 |
| Pakistan | 4 | 0.004 |
| Spain | 3 | 0.003 |
| the United Kingdom | 3 | 0.003 |

Supplementary Table 1 Top 15 countries by productive corresponding authors in research on TCM treatment of MAFLD (Scopus)
